# A family of splice variants of CstF-64 expressed in vertebrate nervous systems

**DOI:** 10.1186/1471-2199-10-22

**Published:** 2009-03-12

**Authors:** Ganesh S Shankarling, Penelope W Coates, Brinda Dass, Clinton C MacDonald

**Affiliations:** 1 Department of Cell Biology and Biochemistry, Texas Tech University Health Sciences Center, Lubbock, Texas, 79430-6540, USA

## Abstract

**Background:**

Alternative splicing and polyadenylation are important mechanisms for creating the proteomic diversity necessary for the nervous system to fulfill its specialized functions. The contribution of alternative splicing to proteomic diversity in the nervous system has been well documented, whereas the role of alternative polyadenylation in this process is less well understood. Since the CstF-64 polyadenylation protein is known to be an important regulator of tissue-specific polyadenylation, we examined its expression in brain and other organs.

**Results:**

We discovered several closely related splice variants of CstF-64 – collectively called βCstF-64 – that could potentially contribute to proteomic diversity in the nervous system. The βCstF-64 splice variants are found predominantly in the brains of several vertebrate species including mice and humans. The major βCstF-64 variant mRNA is generated by inclusion of two alternate exons (that we call exons 8.1 and 8.2) found between exons 8 and 9 of the CstF-64 gene, and contains an additional 147 nucleotides, encoding 49 additional amino acids. Some variants of βCstF-64 contain only the first alternate exon (exon 8.1) while other variants contain both alternate exons (8.1 and 8.2). In mice, the predominant form of βCstF-64 also contains a deletion of 78 nucleotides from exon 9, although that variant is not seen in any other species examined, including rats. Immunoblot and 2D-PAGE analyses of mouse nuclear extracts indicate that a protein corresponding to βCstF-64 is expressed in brain at approximately equal levels to CstF-64. Since βCstF-64 splice variant family members were found in the brains of all vertebrate species examined (including turtles and fish), this suggests that βCstF-64 has an evolutionarily conserved function in these animals. βCstF-64 was present in both pre- and post-natal mice and in different regions of the nervous system, suggesting an important role for βCstF-64 in neural gene expression throughout development. Finally, experiments in representative cell lines suggest that βCstF-64 is expressed in neurons but not glia.

**Conclusion:**

This is the first report of a family of splice variants encoding a key polyadenylation protein that is expressed in a nervous system-specific manner. We propose that βCstF-64 contributes to proteomic diversity by regulating alternative polyadenylation of neural mRNAs.

## Background

Alternative mRNA processing is the mechanism by which multiple forms of mRNAs are generated from a common pre-mRNA via differential ligation of exons (alternative splicing, [1]), or differential 3' end site choice (alternative polyadenylation, [2]). Alternative mRNA processing contributes to proteomic diversity by generating protein isoforms that have different biochemical and structural properties [3,4]. Both of these processes are regulated in a tissue-specific manner, with the highest incidence occurring in the nervous system [5-7]. Not surprisingly, the processes of splicing and polyadenylation are coupled, leading to a high degree of interaction between the two processing machineries [8,9]. However, there have been only a few reports of alternative processing of mRNAs encoding critical proteins of the polyadenylation machinery: alternative splicing of poly(A) polymerase (PAP, [10]) and alternative splicing and polyadenylation of the 77 kDa subunit of the cleavage stimulation factor (CstF-77) in Drosophila [11] and mammals [12] have been reported. Alternatively spliced forms of these and other polyadenylation proteins could contribute to expansion of the proteome by promoting tissue-specific polyadenylation.

Like splicing, polyadenylation is an essential step in gene expression. The process of 3' end formation promotes transcription termination, mRNA export from the nucleus to the cytosol, mRNA stability, and translation initiation [13,14]. In addition to having a constitutive role in gene expression, polyadenylation also contributes to the generation of mRNA isoforms in a tissue-specific manner, particularly in the nervous system. For example, the mRNAs encoding β-adducin [15], huntingtin [16], amyloid precursor protein [17], ferritin heavy chain [18,19], glutamate transporter EAAT2 [20], voltage-gated potassium channel [21], 68-kDa neurofilament [22], and many other proteins [23-28] have alternative polyadenylation signals that lead to production of nervous system-specific transcripts. Nervous system-specific coupling of alternative splicing and polyadenylation of mRNAs encoding calcitonin/calcitonin gene-related peptide [29], neural cell adhesion molecule [30], and vesl-1/homer1 [31] proteins lead to generation of tissue-specific protein isoforms from a common pre-mRNA. It has been speculated that use of alternative polyadenylation signals in these mRNAs may be due to expression of nervous system-specific polyadenylation factors [32], although none have been reported. CstF-64 is a key subunit of the polyadenylation complex that is known to function in regulation of polyadenylation [33,34]. Important domains of CstF-64 include an RNA-binding domain [35-38], CstF-77 interaction domain [39], proline/glycine-rich domain [36], MEARA repeat domain [40], and a conserved C-terminal domain [41]. Furthermore, a paralogous form of CstF-64, τCstF-64, was previously discovered in mouse male germ cells [42] and was found to be essential for normal spermatogenesis [43].

In this paper, we report the discovery of a family of alternatively spliced forms of the CstF-64 mRNA that are expressed in the nervous system of all vertebrate animals we have examined. These splice variants, which we collectively call βCstF-64, are due to inclusion of one or two alternate exons between constitutive exons 8 and 9. In mice, these alternate exons join to an alternative 3' splice site within exon 9. Mice express another minor splice variant, αCstF-64, which is formed by joining of exon 8 to the alternative 3' splice site in exon 9. However, the αCstF-64 splice variant was not observed in any other species including rats or humans. All the βCstF-64 splice variants are in-frame with the CstF-64 coding region and encode up to 49 additional amino acids in the proline/glycine-rich domain. The βCstF-64 splice variants are expressed in brains of many vertebrate species, including human and turtle, and the genome of simple vertebrates such as pufferfish and zebrafish contain homologous sequences. This leads us to hypothesize that the βCstF-64 variants have an important evolutionarily conserved role in brain function. Other experiments suggest that βCstF-64 is expressed predominantly in neurons, suggesting that it plays a role in expression of alternatively polyadenylated mRNAs important for neuronal function, thus contributing to proteomic diversity in the nervous system.

## Results

### Alternatively spliced CstF-64 mRNAs are present in mouse brain

**Figure 1:**
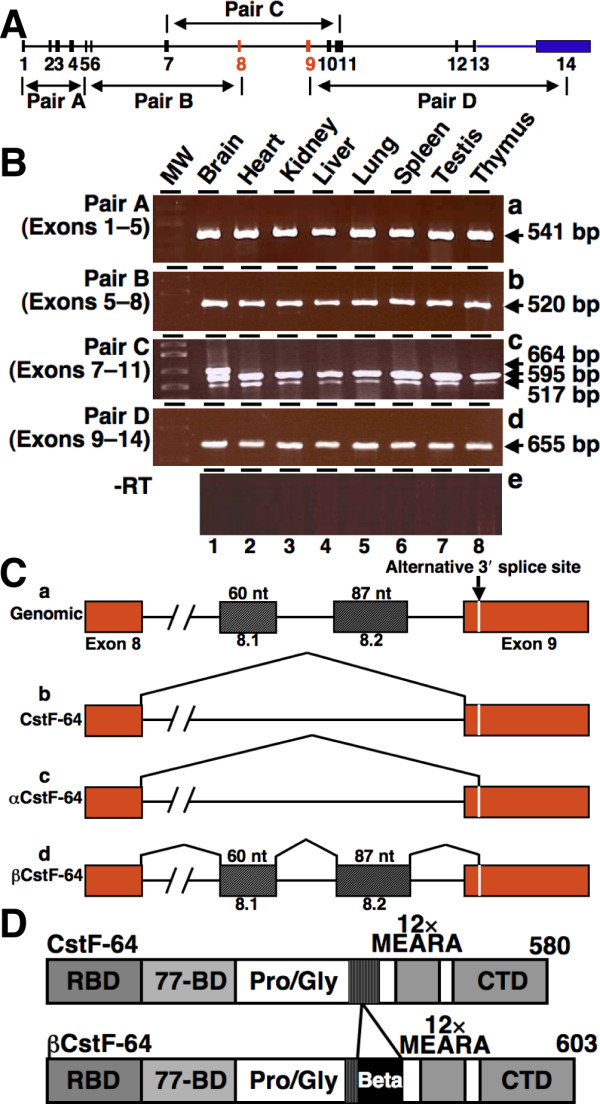
**An alternatively spliced mRNA of CstF-64 is present in mouse brain**. **A) **Gene structure of CstF-64 and location of primer pairs. Boxes indicate exons; black line indicates introns; exons 8 and 9 are in red while all the other exons are black; the 3' UTR is blue. **B) **RT-PCR analysis of alternatively spliced mRNAs of CstF-64. RNA from indicated mouse tissues was subjected to RT-PCR using primer pairs A, B, C, and D (panels **a**, **b**, **c**, and **d **respectively). Sizes of amplified products are indicated at the right. Panel **e **(-RT) denotes RT-PCR using primer pair C with no reverse transcriptase added. **C) **Splicing patterns in CstF-64 mRNA. **a) **CstF-64 genomic structure showing exon 8, 9 and the intervening intron. Boxes indicate exons and horizontal lines indicate introns; vertical lines indicate splicing patterns. The thin white line in exon 9 denotes the alternative 3' splice site. **b) **Splicing pattern of the regular form of CstF-64 mRNA. **c) **Splicing pattern of the shorter CstF-64 mRNA (αCstF-64). **d) **Splicing pattern of βCstF-64 mRNA. The sizes of the βCstF-64-specific exons (8.1 and 8.2) are indicated. **D) **Predicted domain structures of mouse CstF-64 and βCstF-64. Shown are the RNA-binding domains (RBD, dark gray), the region of interaction with CstF-77 (77-BD, light gray), the proline/glycine domain (Pro/Gly, white box), MEARA repeat domain (12× MEARA, dark gray) and the conserved C-terminal domain (CTD, light gray). The region within the Pro/Gly domain that contains a deletion of 26 amino acids is indicated by vertical lines, and the 49 amino acid βCstF-64-specific domain is indicated by the black box.

**Figure 2:**
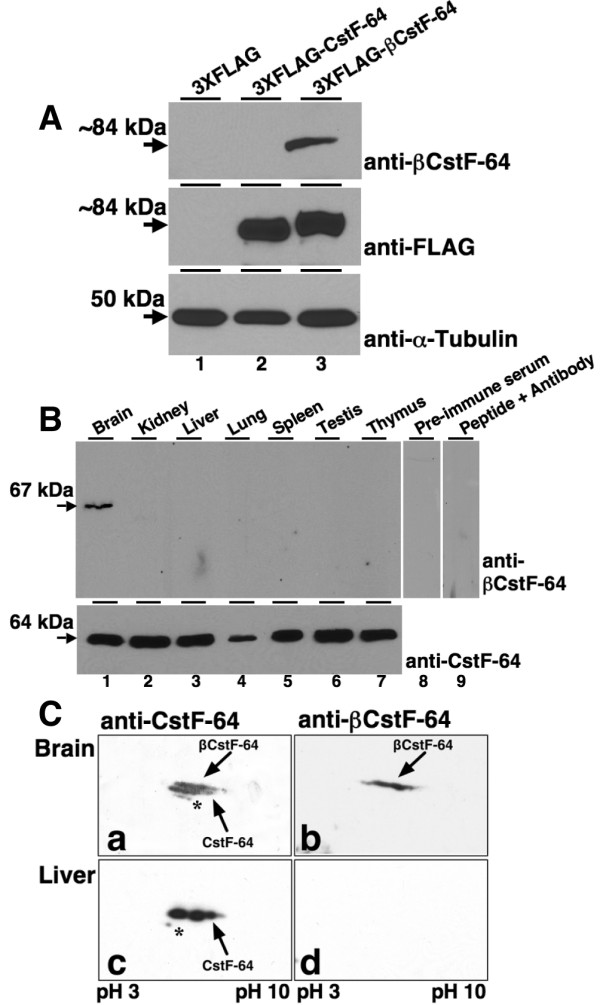
**The βCstF-64 protein is expressed predominantly in mouse brain**. **A) **Whole cell extracts from HeLa cells transfected with 800 ng of either 3XFLAG (lane 1), 3XFLAG-CstF-64 (lane 2) or 3XFLAG-βCstF-64 (lane 3) were subjected to immunoblot analysis using anti-βCstF-64 (upper panel), anti-FLAG (middle panel), or anti-α-Tubulin (lower panel) antibodies. The apparent molecular weights of 3XFLAG-CstF-64, 3XFLAG-βCstF-64, and tubulin are indicated at left. **B) **Protein immunoblot detection of βCstF-64 in various mouse tissues. Nuclear extracts from indicated mouse tissues were subjected to immunoblot analysis using pre-immune serum (lane 8), peptide-blocked anti-βCstF-64 antibody (lane 9), anti-βCstF-64 antibody (upper panel, lanes 1–7), or anti-CstF-64 antibody (lower panel, lanes 1–7). The apparent molecular weights of CstF-64 and βCstF-64 are indicated at left. **C) **2D-PAGE analysis of mouse brain and liver nuclear extracts. Nuclear extracts were resolved in the first dimension by isoelectric focusing on a pH 3–10 immobiline strip as indicated. The proteins were then resolved in the second dimension by denaturing PAGE, transferred to nitrocellulose membrane and probed with the anti-CstF-64 antibody (panels **a **and **c**) or anti-βCstF-64 antibody (panels **b **and **d**). The arrows denote CstF-64 and βCstF-64. The asterisk in panels **a **and **c **indicates a pattern that may be αCstF-64.

Previous results from our laboratory indicated that CstF-64 mRNA could be subject to alternative splicing (B. Dass and A. M. Wallace, unpublished). In order to investigate this possibility, we conducted an RT-PCR survey of CstF-64 mRNA in mouse tissues using primers that together span the entire CstF-64 coding region (Figure 1). RNA was isolated from various mouse tissues as indicated and subjected to RT-PCR using primer pairs A, B, C and D that span exons 1–5, 5–8, 7–11 and 9–14 respectively (Figure 1A). RT-PCR with primer pairs A, B, and D yielded a single PCR product in all tissues that corresponded to the reported form of CstF-64 (Figure 1B, panels **a**, **b**, **d**, lanes 1–8). This suggests little or no alternative splicing between exons 1–8 and 9–14. However, RT-PCR using primer pair C (which spans exons 7–11) resulted in multiple PCR products (Figure 1B, panel **c**). Every mouse tissue examined showed the presence of the reported form of CstF-64 (595 bp) and a shorter splice variant that we named αCstF-64 (panel **c**, lanes 1–8). Cloning and sequencing of the αCstF-64 PCR product indicated that the shorter splice variant of CstF-64 was generated by joining of exon 8 to an alternative 3' splice site in exon 9 resulting in an mRNA molecule shorter by 78 nucleotides (Figure 1C). We reported this alternatively spliced form to GenBank as αCstF-64, variant 4 (GenBank: EU616681). The αCstF-64 splice variant is in frame with exon 9, and should result in a protein isoform that is shorter by 26 amino acids (not tested, but see Figure 2C). Since the αCstF-64 splice variant was not expressed in any other species we examined (see below), we chose not to characterize it further.

Surprisingly, using primer pair C, we found that brain had a larger PCR product (664 bp) that was absent from all the other tissues (Figure 1B, panel **c**, compare lane 1 to lane 2–8). Cloning and sequencing of the larger PCR product revealed that it contained an additional 147 nucleotides between exon 8 and the αCstF-64 splice site within exon 9 (see Additional File 1 for nucleotide sequence). We named the alternate exons 8.1 and 8.2, and the longer brain-expressed variant CstF-64, βCstF-64. The above form of mouse βCstF-64 containing exons 8.1, 8.2 and use of alternative 3' splice site was given the GenBank nomenclature βCstF-64, variant 1 (GenBank: EU616682.1). We compared the sequence of the βCstF-64 variant region to the mouse genome and determined that βCstF-64 was generated by splicing of two alternate exons (8.1 and 8.2, Figure 1C) from the intronic region between exons 8 and 9 and selection of an alternative 3' splice site in exon 9. Exons 8.1 and 8.2 contained 60 and 87 nucleotides respectively. The alternative splicing patterns suggests that βCstF-64 remains in-frame with the CstF-64 coding region, and that it encodes an expressed protein isoform of CstF-64. The predicted domain structure of the protein isoform encoded by βCstF-64 mRNA is illustrated in Figure 1D; this predicted protein isoform would lack 26 amino acids in the proline/glycine-rich domain of CstF-64 encoded by exon 9 and contain an additional 49 amino acids encoded by exons 8.1 and 8.2 (Figure 1D). BLAST comparison of the brain-expressed βCstF-64 domain to all sequences in GenBank revealed no other described proteins with similar features (not shown). Thus, these data suggest that mouse βCstF-64 mRNA encodes a protein in brain that is 23 amino acids longer than CstF-64, containing a unique 49 amino acid brain-specific domain.

### The βCstF-64 protein isoform is expressed in mouse brain

In order to investigate whether the βCstF-64 protein was expressed in mouse brain, antibodies were raised against 16 amino acids of the unique 49 amino acid domain of the protein (Materials and Methods). The specificity of the anti-βCstF-64 antibody was tested using recombinant βCstF-64 and CstF-64 proteins expressed in HeLa cells (Figure 2A). Plasmids encoding epitope-tagged (3XFLAG)-βCstF-64 and CstF-64 proteins were transfected into HeLa cells as described in "Materials and Methods". Whole cell lysates were prepared and subjected to SDS-PAGE and immunoblot analysis using anti-βCstF-64 (Figure 2A, top panel), anti-FLAG (Figure 2A, middle panel), and anti-tubulin antibodies (Figure 2A, bottom panel). This experiment demonstrated that the βCstF-64 epitope was not detected in HeLa cells (lane 1) or in HeLa cells transfected with FLAG-CstF-64 (lane 2) but was expressed at the predicted size in HeLa cells transfected with FLAG-βCstF-64 (lane 3) suggesting that the anti-βCstF-64 antibody could uniquely distinguish CstF-64 and βCstF-64 in mammalian cells.

Next, expression of βCstF-64 protein in various mouse tissues was investigated using the anti-βCstF-64 antibody. Nuclear extracts from the indicated mouse tissues were subjected to immunoblot analysis using the anti-βCstF-64 and anti-CstF-64 antibodies (Figure 2B). Using the anti-CstF-64 antibody, CstF-64 was detected in all tissues (Figure 2B, lower panel). In contrast, using the anti-βCstF-64 antibody, a protein of approximately 68 kDa was detected in brain but not in other tissues examined (Figure 2B, upper panel, compare lanes 1 and 2–7). Detection of this protein was blocked by preincubation of the antibody with the 16 amino acid peptide that was used as an immunogen (lane 9). These results suggest that while the CstF-64 protein is expressed in most or all tissues [44], the βCstF-64 isoform is restricted to brain and probably other neural tissues.

### βCstF-64 and CstF-64 protein levels are similar in mouse brain

We wanted to investigate the relative levels of the CstF-64 and βCstF-64 proteins in mouse brain. Since we could not resolve βCstF-64 from CstF-64 proteins in a single dimension (Figure 2B and data not shown), 2D-PAGE analysis was employed to investigate this. Nuclear extracts from mouse brain and liver were resolved in the first dimension by isoelectric focusing and in the second dimension by SDS-PAGE [45]. Using the anti-CstF-64 antibody, at least two closely opposed patterns of spots were detected in brain (Figure 2C, panel **a**) while a single pattern of spots was detected in liver (Figure 2C, panel **c**). The basic to acidic range of spots most likely reflects differential phospohorylation of CstF-64 [36,46]. Identical blots were probed with the anti-βCstF-64 antibody, and only a single pattern of spots was detected in brain (Figure 2C, panel **b**), while no signal was detected in liver (panel **d**). Alignment of the anti-CstF-64 blot (panel **a**) with the anti-βCstF-64 blot (panel **b**) confirmed that the anti-βCstF-64 pattern overlapped with the upper pattern in the anti-CstF-64 blot. This suggested that only the upper pattern detected in brain by the anti-CstF-64 antibody contained the βCstF-64 epitope. Judging by the relative intensities of the two patterns of spots in brain (panel **a**), we estimate that the protein levels of CstF-64 and βCstF-64 are approximately the same in mouse brain. Interestingly, a faint pattern of spots is seen in both brain and liver using the anti-CstF-64 antibody (panels **a **and **c**, asterisks). We speculate that this might represent the mouse-specific αCstF-64 protein isoform.

### The βCstF-64 family of splice variants is evolutionarily conserved

**Figure 3:**
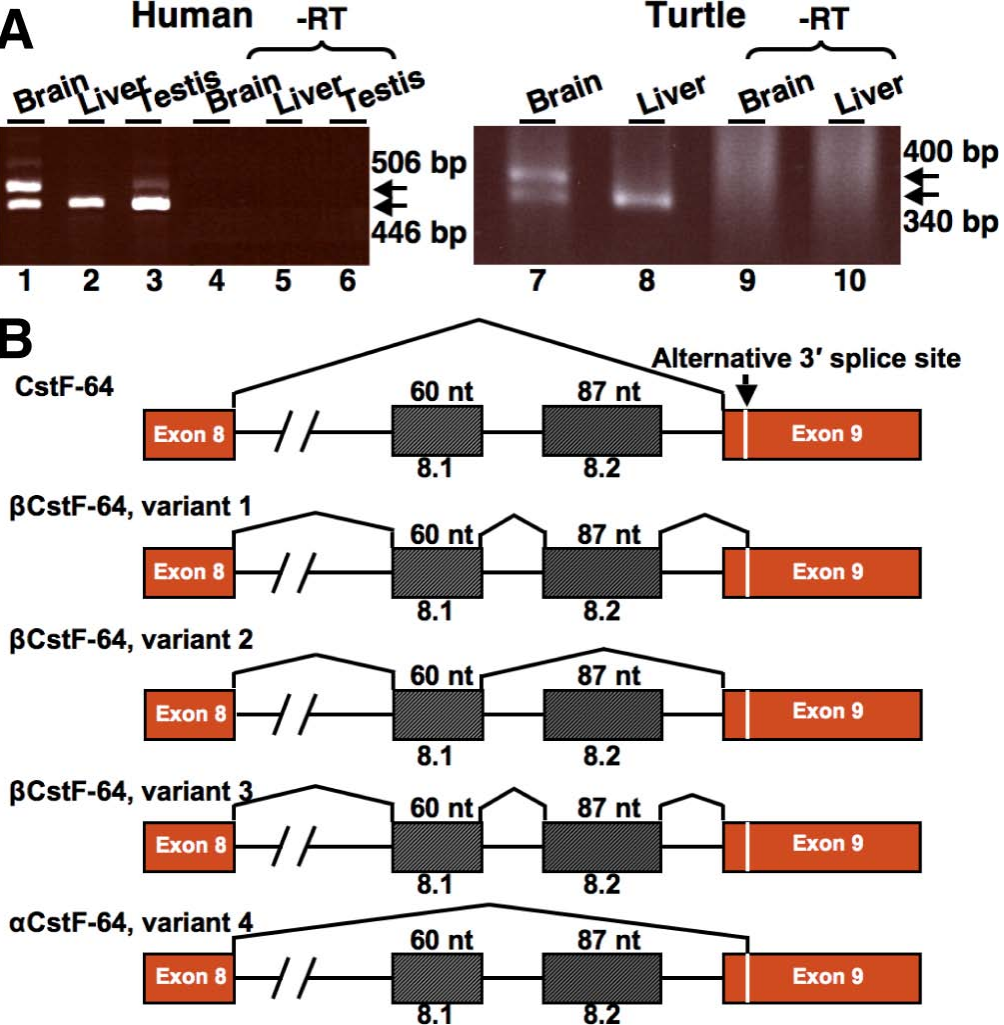
**The βCstF-64 family of splice variants is evolutionarily conserved in vertebrates**. **A) **RT-PCR analysis of human and turtle CstF-64 mRNAs. Human brain, liver, and testis RNAs were subjected to RT-PCR using primers flanking exons 8 and 9 of the human CstF-64 gene (lanes 1–6). RNA isolated from brain and liver of adult turtle was subjected to RT-PCR analysis using primers flanking exons 8 and 9 of the turtle CstF-64 gene (lanes 7–10). RT-PCR with no reverse transcriptase (-RT) is shown in lanes 4–6 (left panel) and lanes 9, 10 (right panel). **B) **Alternative splicing patterns of CstF-64 in various animal species. The splicing pattern of CstF-64 is shown on top. The distributions of the βCstF-64 variant mRNAs in different animal species is as follows: βCstF-64, variant 1: *Mus musculus*; βCstF-64, variant 2: chimpanzee (*Pan troglodytes*), bovine (*Bos taurus*), dog (*Canis familiaris*), thirteen-lined ground squirrel (*Spermophilus tridecemlineatus*), gray short-tailed opossum (*Monodelphis domestica*), African clawed toad (*Xenopus laevis*), duck-billed platypus (*Ornithorhynchus anatinus*), zebrafish (*Danio rerio*), and pufferfish (*Tetraodon nigroviridis*); βCstF-64, variant 3: Algerian mouse (*Mus spretus*), rat (*Rattus norvegicus*), human (*Homo sapiens*), pig (*Sus scrofa domestica*); βCstF-64, variant 4: mouse (*Mus musculus*). The splicing patterns of βCstF-64 mRNA in mice (*Mus musculus *and *Mus spretus*), rat, human, ground squirrel, short-tailed opossum, alligator, and turtle were determined from cloning and sequencing of RT-PCR products. The splicing patterns for chimpanzee, bovine, dog, toad, platypus, zebrafish, and pufferfish were obtained by searches of EST and protein databases utilizing BLASTX and BLASTP programs (not shown).

In order to determine whether βCstF-64 was also expressed in brains of other mammalian species, RT-PCR analysis was conducted using human RNA samples (Figure 3A). RT-PCR analysis using human-specific primers flanking exons 8 and 9 (Table 1) indicated that CstF-64 mRNA was present in brain, liver and testis (Figure 3A, lanes 1–3). A larger PCR product was detected in human brain RNA (Figure 3A, lane 1). Smaller amounts of this product were also detected in human testis (lane 3). Cloning and sequencing of the larger PCR product from human brain revealed a product that contained only exon 8.1 and lacked exon 8.2 and use of the alternative 3' splice site. This demonstrated that the alternatively spliced βCstF-64 isoform in human brain differed from that in mice. We named this isoform, βCstF-64 variant 2 (GenBank: EU616679.1). However, EST database searches (NCBI) showed that mRNAs containing exon 8.1 joined to exon 8.2 were found in humans (Accession numbers: AK095684.1, DA513594.1 and wild boar (Accession number: AJ959057.1). Therefore, using primers specific to the predicted exon 8.2, we detected mRNAs containing this exon in humans (data not shown). We have never observed a βCstF-64 variant mRNA (either by database searches or by cloning) containing exon 8 joined to exon 8.2 in either humans, mice or any other mammals. The βCstF-64 mRNA that contained both exons 8.1 and 8.2 but lacked use of the alternative 3' splice site in exon 9 was given the GenBank nomenclature, βCstF-64 variant 3 (GenBank: EU616680.1). These data suggested that both βCstF-64 variant 2 (exon 8.1 only) and βCstF-64 variant 3 (exon 8.1 and 8.2) are expressed in human brain. The βCstF-64 variant 3 mRNA was also detected in the brains of the mouse species *Mus spretus *and in rats (data not shown). No product corresponding to αCstF-64 was seen in any of the human samples (lanes 1–3).

**Table 1:** Primers used in detection and analysis of CstF-64 splice variants

**Species**	**Primer Name**	**Primer Sequence**
Mouse	Pair A	5' GGGTGAGCCATGGCGGGTT 3' 5' CTGTTCTGGTGGAAGACTGGCAA 3'

Mouse	Pair B	5' CCCCAGGAAGCACGAAACA 3' 5' CCTCGTTCCATGGGCACTG 3'

Mouse, Rat	Pair C	5' CAATGGCGCACCTCCTATGATG 3' 5' GGCACGGGCTTCCAGTCCT 3'

Mouse	Pair D	5' GATTAGATGCACGGGGGATGGA 3' 5' TGGAGCAATGGCGATGTAAGACC 3'

Human	Pair C	5' CCCCTCAGGCCCAGTCTTTG 3' 5' TGGCCCTCCCCTCAGTTCAT 3'

Turtle	Pair C	5' GACAGATGCCAGCCTCCGTAGC 3' 5' CCATTGGTCCTCCCCTCATTTCAT 3'

Mouse	X	5' CAATGGCGCACCTCCTATGATG 3'

Mouse	Y	5' TTCCACCTTGCATGCTTGCTC 3

Mouse	E	5 ' GATCTATGGCGGGTTTGCCAGTGAG 3'

Mouse	F	5 ' TCTAGATCAAGGTGCCCCAGTGGATTTC 3

Nucleotide sequences of the various primer pairs used in this study are indicated at right. The names of primer pairs along with their corresponding species are indicated at left in the table.

We wanted to determine whether the βCstF-64 variant family was restricted to mammals, or could be detected in other vertebrate species. Therefore, RT-PCR analysis was conducted using RNA from brain and liver of turtle (*Trachemys scripta elegans*). RT-PCR products corresponding to βCstF-64 mRNA were detected in turtle brain (Figure 3, lane 7) but not liver (Figure 3, lane 8). Cloning and sequencing of the band revealed that its splicing pattern was similar to βCstF-64 variant 2 mRNA observed in human (Figure 3B). RT-PCR analysis suggested that βCstF-64 mRNA with a splicing pattern similar to that observed in turtle was also present in ground squirrel, alligator, and Monodelphis (not shown, summarized in Figure 3B).

Database searches using exons 8.1 and 8.2 as query were conducted to determine the distribution of βCstF-64 in various animal species (Figure 3B and Additional File 2). Comparison of mouse βCstF-64 nucleotide sequence to the human genome showed that exon 8.1 was 98% identical whereas exon 8.2 was 96% identical. The nucleotide sequence of exons 8.1 and 8.2 were 96% identical when compared to that of rhesus monkey (*Macaca mulatta*). Additionally, survey of the whole-genome-shotgun sequence database at NCBI showed a high degree of conservation of exons 8.1 and 8.2 in the genomes of other mammalian species such as guinea pig, horse, cat, and others. Finally, using BLASTX and BLASTP searches at the NCBI database, amino acids encoded by exon 8.1 were predicted to be present in proteins from chimpanzee, cow, dog (eutherian mammals), platypus (monotreme), Xenopus (amphibian), pufferfish, and zebrafish (fish). We note that exon 8.2 was not explicitly observed in these species, but the incomplete nature of some of these genome data sets does not allow us to rule out its presence. The high degree of conservation in vertebrate animal species suggests an essential function for the βCstF-64 family of splice variants in neural mRNA polyadenylation.

### βCstF-64 mRNA is present throughout the mouse nervous system

**Figure 4:**
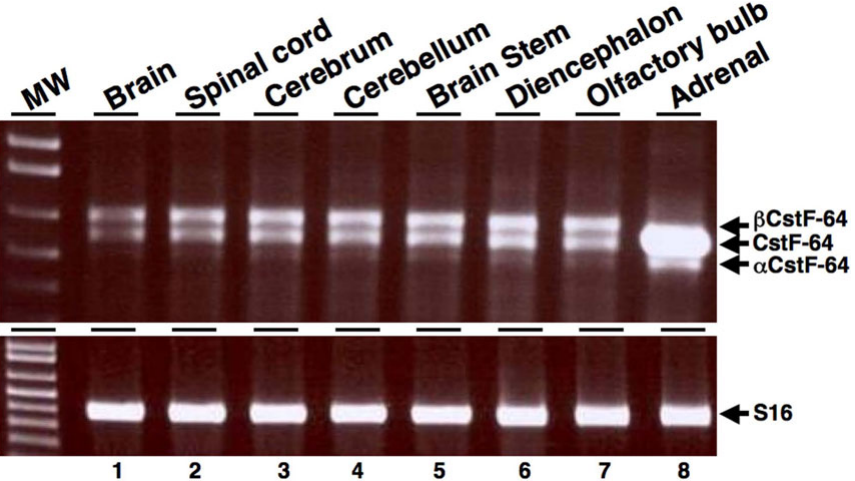
**βCstF-64 mRNA is present throughout the mouse nervous system**. RT-PCR analysis was performed on RNA isolated from the spinal cord, adrenal gland and five regions of mouse brain using primer pair C that flanks exons 7–11 of the CstF-64 gene. RT-PCR products were resolved by 1% agarose gel electrophoresis. RT-PCR of ribosomal S16 mRNA (lower panel) was used a loading control.

An RT-PCR approach was used to investigate the expression of βCstF-64 mRNA in mouse nervous system. RNA was isolated from mouse spinal cord and five different regions of the brain (cerebrum, cerebellum, brain stem, diencephalon and olfactory bulb). βCstF-64 mRNA was detected in all five brain regions (Figure 4, lanes 3–7) and in spinal cord, but not in adrenal gland (Figure 4, lane 8), consistent with nervous system-predominant expression of βCstF-64. RT-PCR of ribosomal S16 mRNA was used to assess RNA loading (Figure 4, lower panel).

### βCstF-64 mRNA is not present in glial cell lines examined

**Figure 5:**
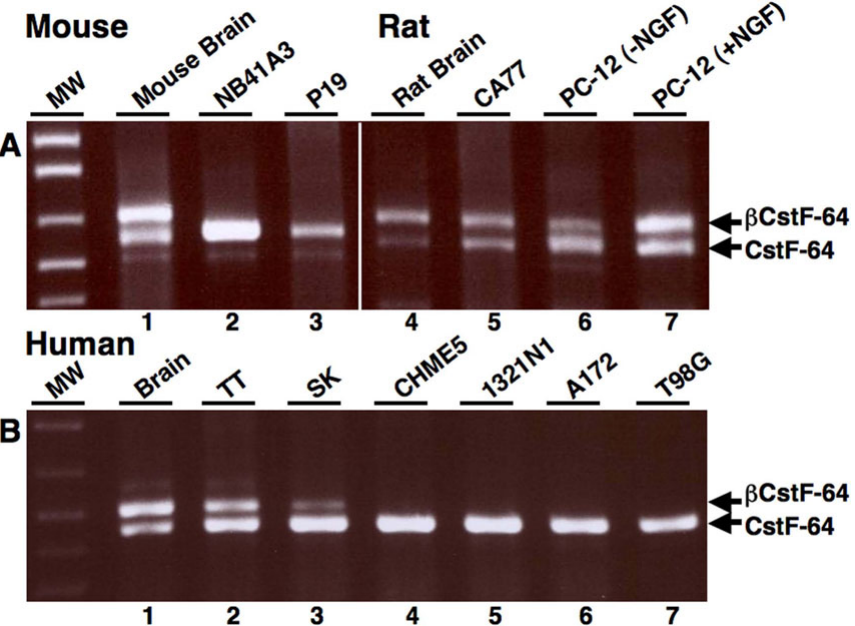
**βCstF-64 mRNA expression in neuronal and glial cell lines**. RT-PCR analysis of βCstF-64 using exon-specific primer pairs (Table S1) from indicated rodent and human neuronal and glial cell lines. **A) **RT-PCR analysis of βCstF-64 mRNA from mouse brain (lane 1), NB41A3 mouse neuroblastoma (lane 2), P19 mouse embryonic carcinoma (lane 3), rat brain (lane 4), CA77 rat thyroid carcinoma (lane 5), PC-12 rat pheochromocytoma (lane 6), or PC-12 rat pheochromocytoma cells treated for 4 days with 50 ng/mL NGF (lane 7). **B) **RT-PCR analysis of βCstF-64 mRNA from human brain (lane 1), TT thyroid carcinoma (lane 2), SK neuroblastoma (lane 3), CHME5 microglia (lane 4), 1321N1 astrocytoma (lane 5), A172 astrocytoma (lane 6), or T98G glioblastoma cells (lane 7).

We wanted to determine in which neural cell types (neurons or glia) βCstF-64 was expressed. For this, RNAs from various rodent and human neuronal and glial cell lines were subjected to RT-PCR using species-specific primers flanking exons 8 and 9 of the CstF-64 gene (Figure 5). The rat and human thyroid carcinoma cell lines (CA77, Figure 5A, lane 5 and TT, Figure 5B, lane 2) used in this study have a neuronal phenotype and are neuroendocrine in nature [47,48]. The PC-12 cell line (Figure 5A, lane 6) derived from rat adrenal chromaffin cells, is neuroendocrine in nature, and, when treated with nerve growth factor (NGF, lane 7), differentiates into a cell that resembles the sympathetic neurons in the peripheral nervous system [49]. βCstF-64 mRNA was detected in CA77 (Figure 5A, lane 5), undifferentiated and NGF-differentiated PC-12 cell lines (Figure 5, lanes 6 and 7), TT (Figure 5B, lane 2), and SK neuroblastoma cell lines (Figure 5B, lane 3). Interestingly, the expression of βCstF-64 mRNA appeared to increase in PC-12 cells treated with nerve growth factor (NGF, lane 7). βCstF-64 mRNA was not detected in mouse P19 embryonic carcinoma cell line (Figure 5A, lane 3), and NB41A3 neuroblastoma cell line (Figure 5A, lane 2). In contrast, βCstF-64 was not detected in any of the human glial cell lines tested (Figure 5B, lanes 4–7), suggesting that βCstF-64 expression might be restricted to neurons in the nervous system.

### βCstF-64 mRNA is present in mice of all ages

**Figure 6:**
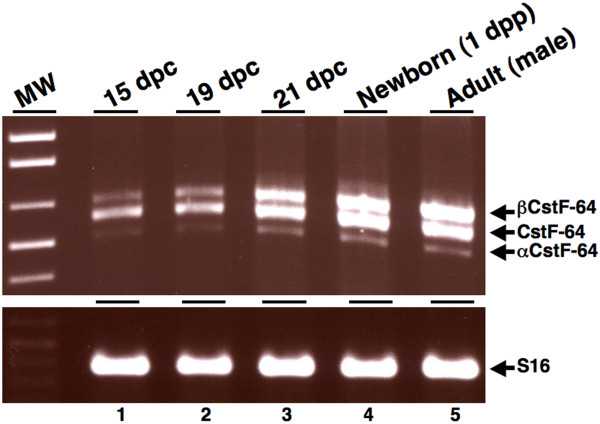
**βCstF-64 mRNA is present in mice of all ages**. RT-PCR analysis using RNA isolated from brain of day 15 embryo (lane 1), day 19 embryo (lane 2), day 21 embryo (lane 3), newborn mice (1 dpp, lane 4), and adult male mice (lane 5). RT-PCR of ribosomal S16 mRNA (lower panel) was used a loading control.

We wanted to investigate the developmental expression of βCstF-64 in mice (Figure 6). For this experiment, female mice mated with male mice were examined for the presence of copulatory plugs. The day copulatory plugs were found, embryos were designated as day 1 post-coitum (1 dpc). Newborn mice were designated as day 1 postpartum (1 dpp) and mice at 42 dpp were designated as adult. RNA from the brains of embryos at 15, 19, 21 dpc, 1 dpp, and adult male mice was subjected to RT-PCR analysis using primers flanking exons 8 and 9 of the CstF-64 gene. The mRNAs corresponding to αCstF-64, CstF-64 and βCstF-64 were detected at 15, 19, and 21 dpc, 1 dpp, and adult mice (Figure 6, lane 1–5). RT-PCR of ribosomal S16 mRNA was used to assess RNA loading (Figure 6, lower panel). βCstF-64 mRNA was also detected in male and female mice of ages 1 dpp, 5 dpp, 7 dpp, 9 dpp, 13 dpp, 15 and 42 dpp at approximately equal levels as CstF-64 mRNA (data not shown).

## Discussion and conclusion

While surveying the CstF-64 mRNA for evidence of alternative splicing, we made the discovery of a family of alternatively spliced forms, collectively called βCstF-64, that were found exclusively in the brain and spinal cord of mice and other vertebrate animal species (Figures 1, 3). In mice, the predominant form of these splice variants includes two alternate exons (8.1 and 8.2) joined to an alternative 3' splice site in exon 9, encoding an additional 49 amino acids while deleting 26 amino acids from exon 9. Interestingly, a smaller splice variant, αCstF-64, which was expressed widely in mice (Figure 1B), was not observed in non-rodent species, suggesting it arose independently of the βCstF-64 variants. We summarized the nomenclature of these isoforms in Figure 3B. Since our data demonstrate that the βCstF-64 protein is expressed in mice (Figure 2), and that the βCstF-64 mRNA family is present in all vertebrate species examined, we propose that the βCstF-64 protein variant has an ancient and essential role in neural function. We hypothesize that βCstF-64 regulates alternative polyadenylation in these tissues, leading to greater diversity of neural gene expression.

A survey of the EST and protein databases (BLASTX and BLASTP programs) indicated that exon 8.1 was predicted to be present in mammalian and non-mammalian genomes. In contrast, a similar analysis showed that exon 8.2 was predicted to be present only in mammalian genomes. This suggests the possibility that exon 8.2 appeared after the divergence of mammals and other vertebrates, and might serve to supplement the function of the more ancient exon 8.1. We also found that the intronic regions between exons 8.1, 8.2 and 9 were highly conserved between mice and humans (84% identity). Since the intronic regions and exons 8.1 and 8.2 likely contain splicing regulatory sequences [50], this supports the notion that the region between exons 8 and 9 is a "hot spot" for alternative splicing during evolution. We failed to detect βCstF-64 exonic sequences in invertebrates such as Drosophila [51] and the simple chordate *Ciona intestinalis *[52], leading us to hypothesize that βCstF-64 has a role in aspects of mRNA polyadenylation that are specific to higher vertebrates, perhaps being critical for important features of neural functions such as myelination [53].

In surveying rodent and human neuronal and glial cell lines (Figure 5), we obtained data suggesting that βCstF-64 expression was restricted to neurons in the nervous system. Even though we did not detect βCstF-64 in any of the glial cell lines examined, we do not rule out the possibility that it might be expressed in normal glial cells. Detailed immunohistochemical analysis of brain slices will provide a better understanding of βCstF-64 expression in neurons and glia.

We obtained data suggesting that βCstF-64 was expressed in cell lines that represent neural progenitor cells and are neuroendocrine in nature (Figure 5). For example, we found evidence for βCstF-64 expression in neural-crest derived cell lines such as CA-77 and thyroid carcinoma cells. We also obtained data suggesting that while βCstF-64 was expressed in undifferentiated and NGF-differentiated PC-12 cell lines (Figure 5), it was not expressed in adrenal gland (Figure 4). PC-12 cells, unlike adrenal chromaffin cells, are thought to possess the pluripotency of primitive progenitor cells such as neural crest cells. These data support the hypothesis that βCstF-64 is important for neuronal gene expression.

What might be the function of βCstF-64 in the nervous system? Our finding that βCstF-64 was expressed throughout the nervous system and at different stages of development suggests that it might be important for global neural gene expression and brain function. Several studies have indicated that many neural mRNAs have multiple polyadenylation sites in their 3' UTRs or use sites that differ from those in other tissues. Many of these mRNAs have regulatory elements surrounding the alternative polyadenylation sites. Differential use of polyadenylation sites within the 3' UTR has been shown to regulate translation [17], stability [54], and rate of transcription [54] of these neural mRNAs. It has been hypothesized that tissue-specific use of polyadenylation sites in these mRNAs might be due to the expression of nervous system-specific polyadenylation factors [15]. Our discovery of the nervous system-specific βCstF-64 splice variant supports the above hypothesis, and leads us to propose that βCstF-64 might recognize alternative polyadenylation signals in these mRNAs contributing to their neural-specific expression. It is possible that βCstF-64 might also contribute to the generation of protein isoforms specific to the nervous system by use of internal poly(A) sites found in many neural mRNAs [29-31].

How might βCstF-64 function in neural mRNA polyadenylation? In one model, βCstF-64 might recognize RNA sequence elements that are specific to nervous system-expressed polyadenylation sites. A similar model has been proposed for the testis-expressed τCstF-64 variant [35,55]. A bioinformatic survey revealed that the downstream U/GU-rich sequences in the pre-mRNA are enriched in nervous system-specific transcripts, lending plausibility to this model [6]. How might βCstF-64 recognize these variant RNA sequence elements if its RNA-binding domain is the same as CstF-64? The insertion in βCstF-64 in the proline/glycine-rich domain might alter the conformation of that domain which might in turn affect the conformation of the RNA-binding domain and alter the RNA binding specificities of βCstF-64 or otherwise affect the overall rate of polyadenylation [56]. In another model, the insertion of the βCstF-64 domain might alter the overall structure of the CstF complex (for example, its dimerization, [57]), affecting its interaction with CPSF or other components of the polyadenylation machinery.

For a final model, we note that the 49 amino acid βCstF-64 domain is not similar to any other known protein domains (data not shown). Based on this, we speculate that the βCstF-64 domain might be a previously undescribed protein-protein interaction domain. It is possible that βCstF-64 interacts with as-of-yet unknown nervous system-specific polyadenylation factors or brings about new interactions within the polyadenylation complex. It might also interact with the splicing machinery since splicing factors are known to modulate polyadenylation efficiency [58-60]. Any of these proposed interactions could help βCstF-64 in generating protein isoforms via recognition of internal intronic and exonic poly(A) sites thus increasing proteomic diversity in the nervous system.

## Methods

### Animals and RNA Samples

All animal studies were conducted in accordance with the National Institutes of Health guidelines and all protocols were approved by the TTUHSC Institutional Animal Care and Use Committee. Tissues from adult male and female CD-1 mice (Charles River Laboratories, Wilmington, DE) were dissected post-euthanasia and stored in RNAlater (Ambion, Austin, TX). Brains from mouse embryos at days 15, 19, and 21 days post-coitus (dpc) were dissected from the gravid females post-euthanasia. The first day on which a vaginal plug was observed in the female, after mating, was considered as 1 dpc. Human brain, liver and testis RNA samples purchased from Ambion (Austin, TX) were from single individuals. Slider turtle (*Trachemys scripta elegans*) brain and liver RNA and 13-lined ground squirrel (*Spermophilus tridecemlineatus*) brain RNA were obtained from B. Dass (TTUHSC, Lubbock, TX) and Alligator (*Alligator mississippiensis*) custom cDNA library was a gift from Phil Hartig (US-EPA). Monodelphis tissues were provided by Janice MacRossin and John L. VandeBerg (Southwest Foundation for Biomedical Research).

### RNA Analysis and RT-PCR

Different regions of the brain and indicated mouse tissues were dissected, total RNA extracted by using TRIzol reagent (Invitrogen, Carlsbad, CA), and treated with TurboDNase (Ambion). Equal amounts of DNased RNA (4 μg) from each tissue sample were used to synthesize cDNA using SuperScriptII Reverse Transcriptase and oligo-dT according to the manufacturer's protocol (Invitrogen). Polymerase chain reaction (PCR, 30 cycles) was conducted using the indicated primers (see Table 1) in an Air Thermocycler (Idaho Technologies, Salt Lake City, UT). Amplicons were resolved by electrophoresis on a 1% agarose gel electrophoresis. Ethidium bromide stained RT-PCR products were excised from the gel (Qiagen, Valencia, CA), cloned using Topo II system (Invitrogen), and identified by DNA sequence analysis.

### Cloning and Plasmids

The plasmid encoding full length βCstF-64 fused to the FLAG epitope tag [61] (3XFLAG-βCstF-64) was created as follows: mouse brain cDNA was subjected to RT-PCR using primers X and Y containing HinDIII, KpnI restriction sites (Table 1) and ligated into a 3X-HA vector encoding full length CstF-64 (kind gift of K. W. McMahon) digested by the same enzymes. Note that this vector also contained the MS2 RNA binding domain though that did not interfere with its expression or antigenicity. The 3XFLAG-βCstF-64 plasmid was created by amplifying full length βCstF-64 using primers E and F containing XbaI, BglII sites (Table 1) using 3X-HA-βCstF-64 as template. This fragment was digested with Xba I and Bgl II, and ligated into a similarly digested 3XFLAG-CstF-64 vector. The cloning scheme of 3XFLAG-CstF-64 is described in [61].

### Cell Culture and Transfections

HeLa cells were grown at 37°C in 5% CO_2 _in Dulbecco's Minimal Eagle Media (CellGro, Manassas, VA) containing 10% cosmic calf serum and 1% penicillin/streptomycin (Gibco). HeLa cells were seeded in 6-well plates at a density of 300,000 cells/well and transfected the next day with 800 ng of the indicated plasmids using Lipofectamine (Invitrogen). Cells were harvested 48 hours post-transfection by rinsing with ice-cold PBS, and lysed in SDS loading buffer [62].

### Antibodies

The following antibodies were used in this study: anti-α-tubulin and anti-FLAG antibodies (Sigma, St. Louis, MO); anti-CstF-64 antibody (3A7, [44]) and anti-βCstF-64 custom antibody generated in rabbits by Genscript Corporation (Piscataway, NJ) by injection of the peptide GPAGPASIERVQGQRT representing part of the βCstF-64 insert.

### Protein Analysis

For protein analysis, nuclear extract was prepared from indicated mouse tissues [63], boiled and sonicated in SDS loading buffer [62]. Protein concentration was measured using the bicinchoninic acid (Pierce, Rockford, IL) assay. Equal amounts of protein from the indicated tissues (20 μg) or HeLa cells were resolved by 10% SDS-PAGE, and transferred to nitrocellulose membranes for immunoblotting. For immunoblotting using anti-CstF-64 antibody, membranes were blocked with Tris-buffered saline containing 0.2% Tween-20 (TBST) with 2% nonfat dry milk (TBST) for 2 hours and treated with anti-CstF-64 antibody [44] at a dilution of 1:50. For immunoblotting using anti-βCstF-64 antibody, the membrane was first treated with 0.2 mM glycine-HCl, pH 2.6 for 30 minutes at room temperature, and rinsed extensively with TBST. Pre-treatment with glycine at low pH was found to enhance the effectiveness of this antibody while decreasing non-specific background. The membrane was then blocked with 2% nonfat dry milk in TBST for 2 hours. The anti-βCstF-64 antibody was used at a dilution of 1:5000 in 2% nonfat dry milk in TBST, 0.05% Empigen (Calbiochem, La Jolla, CA). In the peptide blocking experiment, the anti-βCstF-64 antibody (1 μg/μL) was incubated with 10 fold excess peptide (10 μg) in 2% nonfat dry milk in TBST for 2 hours at room temperature before addition to the membrane. Membranes were subsequently treated with horseradish peroxidase-conjugated goat anti-mouse and anti-rabbit IgG (1:2500) and immunoreactive bands were visualized by chemiluminescence using the Pierce SuperSignal kit (Rockford, IL).

### Two dimensional-PAGE Analysis

Nuclear extracts from mouse brain and liver were prepared as described above. Protein concentration was estimated by BCA assay. 392 μL of rehydration buffer (9 M urea, 2% Triton X-100), 8 μL IPG buffer (pH 4–7) (Amersham Biosciences, Piscataway, NJ) and 0.5 μL 1 M DTT was added to 80 μg of nuclear protein. The mix was layered over a 7 cm pH 3–10 immobiline strip (Amersham Biosciences) and allowed to rehydrate for 16 hours. The strips were then subjected to isoelectric focusing at the following voltages: 200 V for 1 minute, 500 V for 20 minutes, 1000, 1500, 2000, 2500 V for 20 minutes and 3500 V for 60 minutes. The strip was equilibrated in equilibration solution (20% SDS, 50 mM Tris, pH 8.8, 6 M urea, 30% glycerol) containing 65 mM DTT but no iodoacetamide for 15 minutes and then in equilibration solution containing 135 mM iodoacetamide but no DTT for 15 minutes. The strip was then placed horizontally on a 10% SDS gel, subjected to denaturing PAGE and immunoblot analysis.

## Authors' contributions

GSS conducted all experiments except the ones described below and wrote the manuscript. PWC conducted all the dissections described in the paper and helped analyze data. BD provided RNA from slider turtle and sequence information for CstF-64 from various animal species. CCM conceived the study, lent expert guidance, and provided critical comments for developing the manuscript. All authors read and approved the final manuscript.

## Supplementary Material

Additional file 1 **Nucleotide and amino acid sequence of mouse βCstF-64**. The nucleotide sequence of exons 8.1 and 8.2 of mouse βCstF-64 (accession numbers provided) and their corresponding amino acid sequences are indicated. The nucleotide and amino acid sequences of the region deleted from exon 9 via use of the alternative 3' splice site are also shown. Click here for file

Additional file 2 **Multiple sequence alignment of the 50 amino acid βCstF-64 sequence from various animal species**. The amino acid sequences of βCstF-64 from mouse, rat, human, turtle, ground squirrel, alligator and monodelphis were predicted from cloning and in silico translation of RT-PCR products while the rest were determined by searching EST and protein databases at NCBI. Multiple sequence alignment was determined by ClustalW using sequences with the following accession numbers: XP_001068092.1 (Rat), EU616682 (Mouse), EU616679 (Human), AJ959057.1 (Wild boar), XP_529072 (Chimpanzee), Ground Squirrel (B. Dass, unpublished), AAI1265544 (Cow), XP_549135 (Dog), Monodelphis (B. Dass, unpublished), XP_001513073 (Platypus), Alligator (B. Dass, unpublished), Turtle (G. Shankarling and B. Dass, unpublished), NP_001080179.1 (Xenopus), CAG09844.1 (Pufferfish), CU459168.8, CT027817.1 (Zebrafish). Boxed residues denote amino acids that differ from rodent. The various animal species included in this study are indicated on right. Click here for file
