# Supplementary material for: A family of splice variants of CstF-64 expressed in vertebrate nervous systems
Source: BMC Mol Biol. 2009 Mar 12;10:22. doi: 10.1186/1471-2199-10-22 (PMC2660332; doi:10.1186/1471-2199-10-22)
Supplement: Additional file 1 — Nucleotide and amino acid sequence of mouse βCstF-64. The nucleotide sequence of exons 8.1 and 8.2 of mouse βCstF-64 (accession numbers provided) and their corresponding amino acid sequences are indicated. The nucleotide and amino acid sequences of the region deleted from exon 9 via use of the alternative 3' splice site are also shown. [file 1471-2199-10-22-S1.doc]

**Additional files**

**Additional file 1**

**File format: DOC**

**Title: Nucleotide and amino acid sequence of mouse βCstF-64**

**Description:** The nucleotide sequence of exons 8.1 and 8.2 of mouse βCstF-64 (accession numbers provided) and their corresponding amino acid sequences are indicated. The nucleotide and amino acid sequences of the region deleted from exon 9 via use of the alternative 3′ splice site are also shown.

**Nucleotide sequence of exon 8.1 of mouse βCstF-64 mRNA (GenBank: EU616682)**

GAACCTTACAGCACTCGCCCGTGGGACCCGCCGGGCCTGCATCAATTGAGCGAGTTCAAG

**Nucleotide sequence of exon 8.2 of mouse βCstF-64 mRNA (GenBank: EU616682)**

GGCAGAGAACATGGATGATATGGGCATCTGTCCGAGGCTCTACTCCCTCCCTACTGGTGTCAGGAGGCTTGGATGGAATTGCAGTCT

**Amino acid sequence encoded by exon 8.1 of mouse βCstF-64 mRNA**

GTLQHSPVGPAGPASIERVQ

**Amino acid sequence encoded by exon 8.2 of mouse βCstF-64 mRNA**

GQRTWMIWASVRGSTPSLLVSGGLDGIAV

**Nucleotide sequence of the 78 nucleotide region deleted from Exon 9 of mouse CstF-64 (GenBank: EU616681)**

TGCCAATGCAAGACCCGAGAGCAGCTATGCAGCGGGGAGCCTTGCCTACCAACGTCCCAACTCCTCGTGGTCTTTTAG

**Amino acid sequence of the 26 amino acids deleted from Exon 9 of mouse CstF-64**

VPMQDPRAAMQRGALPTNVPTPRGLL

**Additional file 2**

**File format: PDF**

**Title: Multiple sequence alignment of the 50 amino acid βCstF-64 sequence from various animal species**

**Description:** The amino acid sequences of βCstF-64 from mouse, rat, human, turtle, ground squirrel, alligator and monodelphis were predicted from cloning and in silico translation of RT-PCR products while the rest were determined by searching EST and protein databases at NCBI. Multiple sequence alignment was determined by ClustalW using sequences with the following accession numbers: XP_001068092.1 (Rat), EU616682 (Mouse), EU616679 (Human), AJ959057.1 (Wild boar), XP_529072 (Chimpanzee), Ground Squirrel (B. Dass, unpublished), AAI1265544 (Cow), XP_549135 (Dog), Monodelphis (B. Dass, unpublished), XP_001513073 (Platypus), Alligator (B. Dass, unpublished), Turtle (G. Shankarling and B. Dass, unpublished), NP_001080179.1 (Xenopus), CAG09844.1 (Pufferfish), CU459168.8, CT027817.1 (Zebrafish). Boxed residues denote amino acids that differ from rodent. The various animal species included in this study are indicated on right.
