# Supplementary material for: A family of splice variants of CstF-64 expressed in vertebrate nervous systems
Source: BMC Mol Biol. 2009 Mar 12;10:22. doi: 10.1186/1471-2199-10-22 (PMC2660332; doi:10.1186/1471-2199-10-22)
Supplement: Additional file 2 — Multiple sequence alignment of the 50 amino acid βCstF-64 sequence from various animal species. The amino acid sequences of βCstF-64 from mouse, rat, human, turtle, ground squirrel, alligator and monodelphis were predicted from cloning and in silico translation of RT-PCR products while the rest were determined by searching EST and protein databases at NCBI. Multiple sequence alignment was determined by ClustalW using sequences with the following accession numbers: XP_001068092.1 (Rat), EU616682 (Mouse), EU616679 (Human), AJ959057.1 (Wild boar), XP_529072 (Chimpanzee), Ground Squirrel (B. Dass, unpublished), AAI1265544 (Cow), XP_549135 (Dog), Monodelphis (B. Dass, unpublished), XP_001513073 (Platypus), Alligator (B. Dass, unpublished), Turtle (G. Shankarling and B. Dass, unpublished), NP_001080179.1 (Xenopus), CAG09844.1 (Pufferfish), CU459168.8, CT027817.1 (Zebrafish). Boxed residues denote amino acids that differ from rodent. The various animal species included in this study are indicated on right. [file 1471-2199-10-22-S2.pdf]

```
  G T L Q H S P V G P A G P A S I E R V Q - - - - - - - - - - - - - - - - - - - - - - - - - - - - -   Majority
                    10                  20                  30                  40
1 G T L Q H S P V G P A G P A S I E R V Q G Q R T W M I W A S V R G S T P S L L V S G G L D G I A V   Rodent
1 G T L Q H S P V G P A G P A S I E R V Q G Q R T W M I W A S V R G C T P S L L V S G G L D G I A V   Human
1 G T L Q H S P V G P A G P A S I E R V Q G Q R T W M I W A S V R G C T P S L L V S G G L D G I A V   Wildboar
1 G T L Q H S P V G P A G P A S I E R V Q                                                             Chimpanzee
1 G T L Q H S P V G P A G P A S I E R V Q                                                             Squirrel
1 G T V Q H S P V G P T G P A S I E R V Q                                                             Cow
1 G T L Q H S P V G P A G P A S I E R V Q                                                             Dog
1 G S L Q H S P V G P A G P A S I E R V Q                                                             Monodelphis
1 G T L Q H S P V G P T G P A P I E R V Q                                                             Platypus
1 G N L Q L S P V G P A R P A S I E R V Q                                                             Alligator
1 G N L Q L S P V G P A R P A S I E R V Q                                                             Turtle
1 G S L Q H S P V A P A G P T T I E R V P                                                             Xenopus
1 G N L Q H S P T G A S G Q A A I E R P Q                                                             Pufferfish
1 G N L Q D S P V G A A G Q A A I E R S Q                                                             Zebrafish
```

Decoration 'Decoration #1': Box residues that differ from Rodent.
